# *OsProDH* Negatively Regulates Thermotolerance in Rice by Modulating Proline Metabolism and Reactive Oxygen Species Scavenging

**DOI:** 10.1186/s12284-020-00422-3

**Published:** 2020-08-26

**Authors:** Mingxin Guo, Xiaotian Zhang, Jiajia Liu, Linlin Hou, Hanxiao Liu, Xusheng Zhao

**Affiliations:** 1 College of Life Sciences, Luoyang Normal University, Luoyang, 471934 China; 2 Jujube Research Center, Luoyang Normal University, Luoyang, 471934 China

**Keywords:** Rice, *OsProDH*, Proline, Thermotolerance, Reactive oxygen species

## Abstract

**Background:**

Global warming threatens rice growth and reduces yields. Proline plays important roles in plant abiotic stress tolerance. Previous research demonstrated that engineering proline metabolism-related genes can enhance tolerance to freezing and salinity in *Arabidopsis*. *OsProDH* encodes a putative proline dehydrogenase and is a single copy gene in rice. However, whether *OsProDH* plays roles in abiotic stress in rice remains unknown.

**Findings:**

Quantitative RT-PCR analysis revealed that *OsProDH* transcript contents were relatively higher in leaf blade and root tissues and the high temperature treatment repressed expression of *OsProDH*. The predicted OsProDH protein localized in mitochondria. Using the *Oryza sativa ssp. japonica* cultivar KY131, we generated *OsProDH* overexpression (OE) lines and knockout mutant lines using the CRISPR/Cas9 (CRI) system. Overexpression of *OsProDH* decreased proline content, while mutation of *OsProDH* increased proline content compared with that of KY131. The CRI and OE lines were respectively more resistant and sensitive to heat stress than KY131. Heat stress induced proline accumulation and mutation of *OsProDH* led to proline overproduction which reduced H_2_O_2_ accumulation in the seedlings.

**Conclusions:**

*OsProDH* negatively regulates thermotolerance in rice. Our study provides a strategy to improve heat tolerance in rice via manipulating proline metabolism.

## Findings

High temperature stress reduces plant growth and crop productivity, potentially resulting in widespread risk of food insecurity (Battisti and Naylor 2009; Lobell et al. 2011). Declines in yield of crops, such as wheat, maize, and barley, have likely resulted from increases in global temperatures (Lobell and Field 2007). In particular, rice yield has declined by 10% per 1 °C increase during the dry season of crop growth (Peng et al. 2004). Therefore to reduce risks of food insecurity due to rising global temperatures, we must improve modern plant breeding strategies to increase crop tolerance to heat stress by expanding our understanding of the molecular mechanisms underlying plant responses to heat stress and genetic modifications of plants.

Proline is an essential proteinogenic amino acid and plays important roles in plant abiotic-stress tolerance (Nanjo et al. 1999; Székely et al. 2008; Zhang et al. 2017; Liu et al. 2018). To date, much is known about proline synthesis and metabolism in higher plants. Proline is synthesized mainly from glutamate being converted into glutamate-semialdehyde (GSA) by pyrroline-5-carboxylate synthetase (P5CS). Then GSA is spontaneously converted into pyrroline-5-carboxylate (P5C), which is reduced to proline by P5C reductase (P5CR). Proline is degraded into glutamate by two key mitochondrial enzymes: proline dehydrogenase (ProDH) and pyrroline-5-carboxylate dehydrogenase (P5CDH). First, ProDH oxidizes proline into delta^1^-pyrroline-5-carboxylate (P5C) which is subsequently converted into glutamate by P5CDH (Szabados and Savouré 2010). In *Arabidopsis*, the *p5cs1* mutant exhibited a salt-hypersensitive phenotype that led to hyperaccumulation of H_2_O_2_, increased chlorophyll damage and lipid peroxidation (Székely et al. 2008). There are two genes (*AtProDH1* and *AtProDH2*) encoding proline dehydrogenase in *Arabidopsis*. The predicted pre-proteins AtProDH1 and AtProDH2 share 75% identical amino acids (Funck et al. 2010). Antisense suppression of *AtProDH1* led to greater tolerance to freezing and salinity stress in *A. thaliana* (Nanjo et al. 1999). *AtProDH2* was specifically induced during salt stress and promoted proline accumulation under the stress (Funck et al. 2010). So far, much is known about the biological functions of core enzymes involved in proline synthesis and metabolism in *Arabidopsis*. However, little is known about the biological functions of these key enzymes in rice.

In this study, we focused on *OsProDH* (*Os10g0550900*), a single copy gene encoding the putative proline dehydrogenase in rice. We cloned the coding sequence (CDS) and genomic DNA sequence of *OsProDH* by PCR method using the *japonica* variety Kongyu131 (KY131). We compared the CDS with genomic DNA and found four exons in *OsProDH* genomic DNA. The predicted pre-proteins of OsProDH has 454 amino acids and with the proline dehydrogenase domain located at residues 133–436 by searching the NCBI Conserved Domain Database (Additional file 2: Figure S1). Furthermore, alignment of the predicted protein sequences of OsProDH with AtProDH1, AtProDH2, ZmProDH1, ZmProDH2 and SbProDH, showed that these proteins all had proline dehydrogenase domain and high similarity (Additional file 2: Figure S2).

**Fig. 1:**
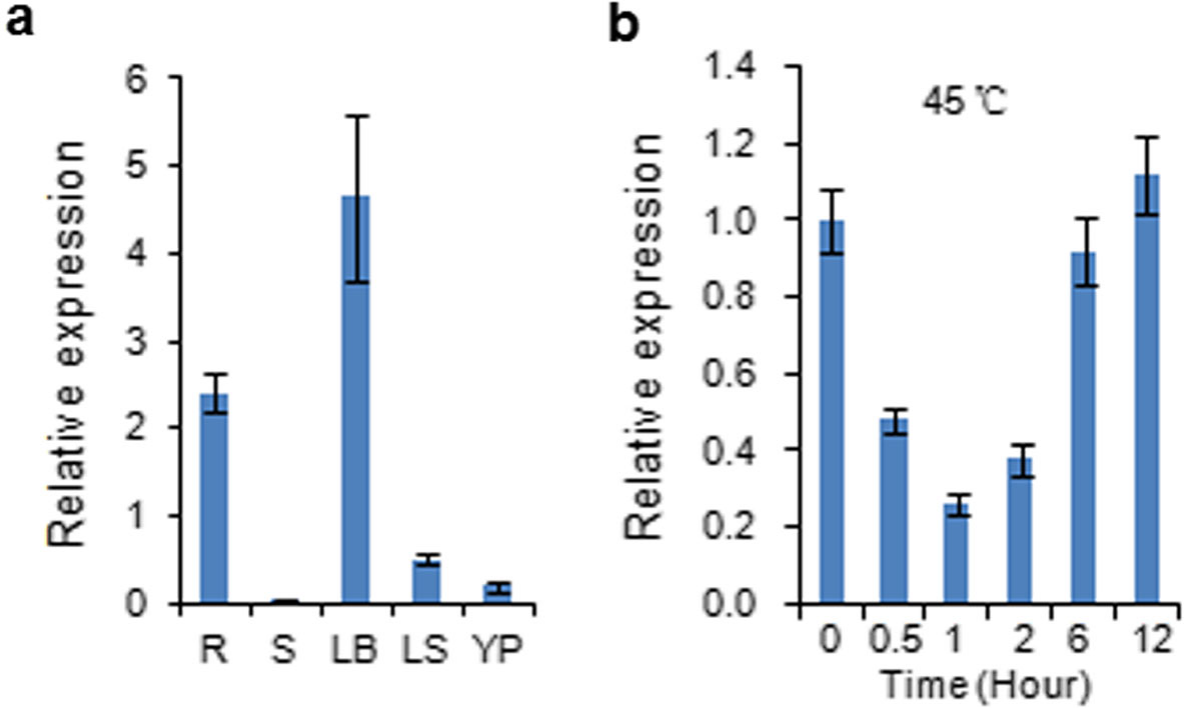
Expresson pattern of *OsProDH*. **a** Tissue-specific expression of *OsProDH* detected by qPCR. R: root; S: stem; LB: leaf blade; LS: leaf sheath; YP: young panicle. Roots were sampled from two-leaf stage seedlings. Stem, leaf blade, leaf sheath were sampled from two-month old plants. Panicle in 5 cm length. The data shown are the mean values of three technical repeats with the SD. **b** Transcriptional response of *OsProDH* to high temperature stress. Two-leaf stage KY131 seedlings were subjected to 45 °C treatment and *OsProDH* expression was determined in the shoots at the indicated time points by qPCR analysis. *OsActin* was used as internal control. The data shown are the mean values of three technical repeats with the SD

Quantitative RT-PCR (qPCR) analysis revealed that *OsProDH* transcripts can be detected in various tissues: root, stem, leaf blade, leaf sheath, and young panicle. Expression levels were relatively higher in leaf blade and root than other tissues (Fig. 1a). To examine the transcriptional response of *OsProDH* to heat stress, two-leaf stage seedlings of KY131 were subjected to 45 °C treatment and the shoots were sampled at 0, 0.5, 1, 2, 6, 12 h after treatment. The results showed that heat stress clearly repressed the expression level of *OsProDH* (Fig. 1b).

**Fig. 2:**
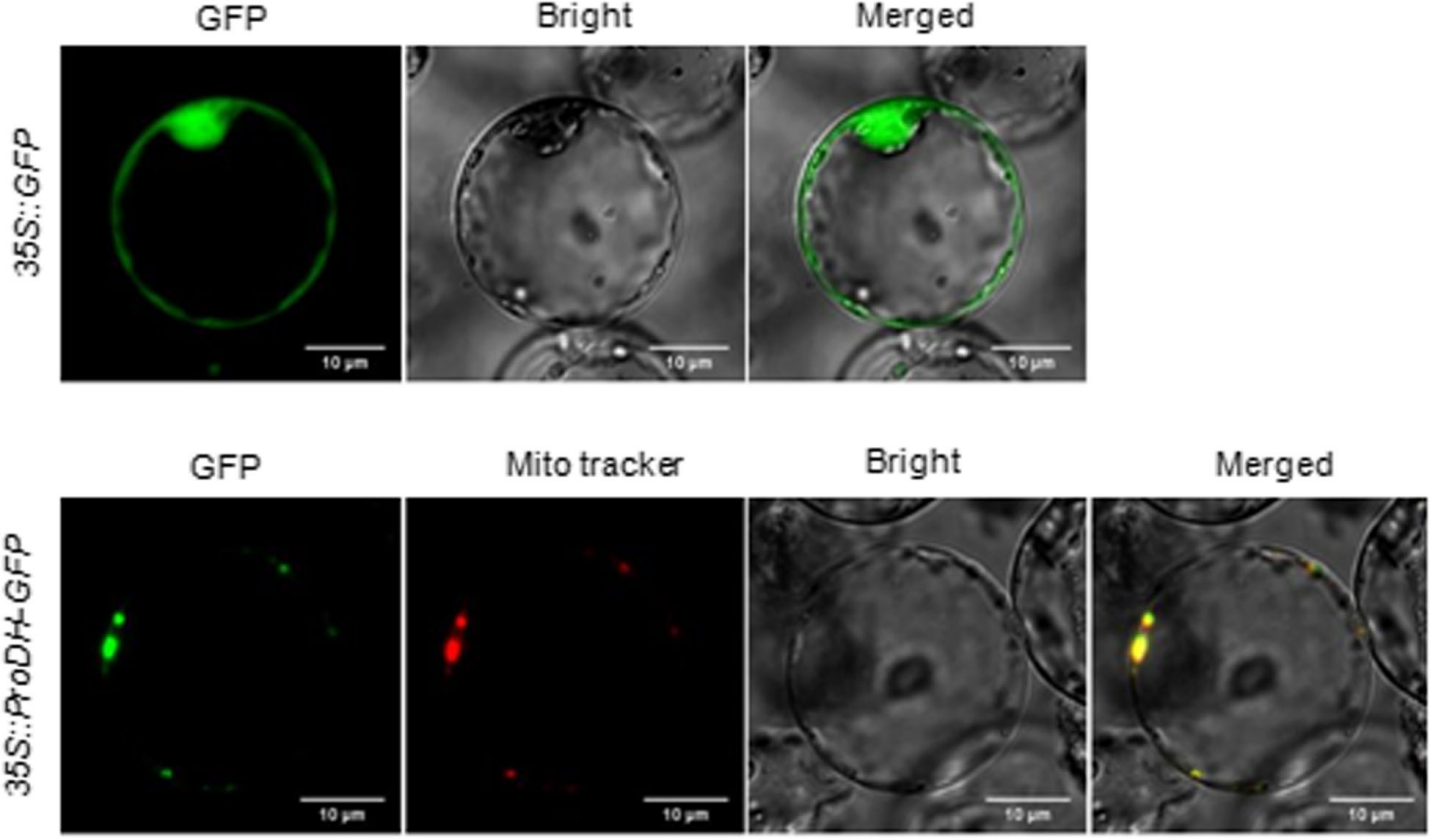
Subcellular localization of OsProDH. GFP and the OsProDH-GFP fusion were transiently expressed in rice protoplasts. Using mitochondrial tracker, it indicated the OsProDH -GFP fusion protein was specifically expressed in the mitochondria

To determine the subcellular localization of OsProDH, a *35S::OsProDH-GFP* vector was introduced into rice protoplasts. As AtProDH1 and AtProDH2 were all localized in mitochondria (Funck et al. 2010), we inferred that the OsProDH might also be located in mitochondria. Using a mitochondrial tracker, we observed that OsProDH-GFP localized in mitochondria, whereas GFP alone localized in the cytoplasm (Fig. 2). Thus results show that OsProDH is a mitochondria-localized protein.

**Fig. 3:**
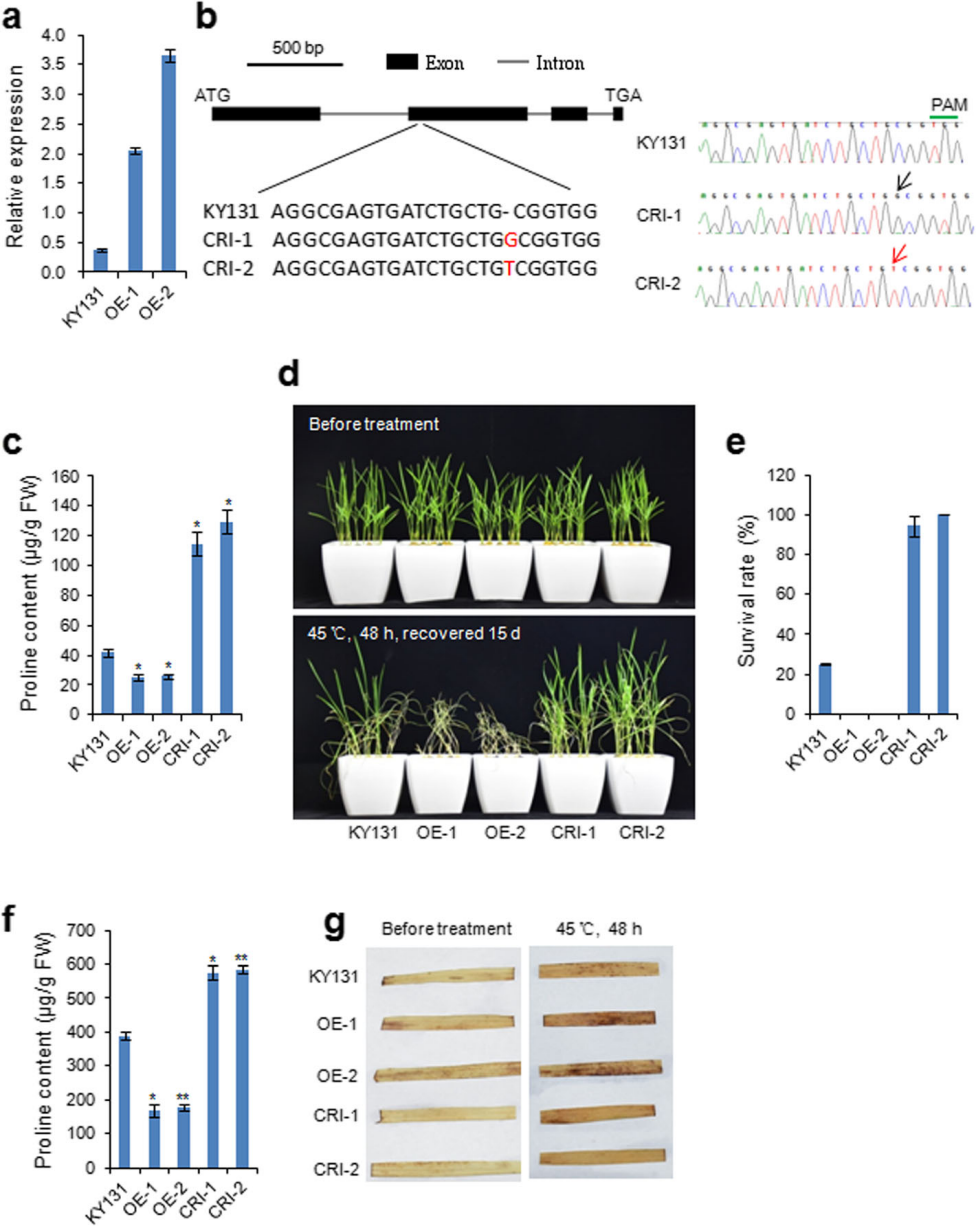
*OsProDH* negatively regulates thermotolerance in rice **a** Transcript levels of *OsProDH* detected by qPCR in KY131 and transgenic two overexpression (OE) lines in shoots at the two-leaf stage rice seedlings. The data shown are the mean values of three technical repeats with the SD. **b** Gene structure of *OsProDH* and sequencing results at target sites in T1 plants produced by CRISPR/Cas9. **c** Proline contents of KY131, OE and CRI lines in shoots at the two-leaf stage rice seedlings under normal conditions. Values are the means ± SE, *n* = 3. Differences between the KY131 and transgenic lines were analyzed with Student’s *t*-test. (*P < 0.05). **d** Phenotypes of KY131, OE and CRI lines under 45 °C treatment. The two-leaf stage seedlings were subjected to high temperature treatment for 48 h and then recovered at normal conditions. **e** Survival rates of KY131, OE and CRI lines after recovering 15 days at normal conditions. (*n* = 3 × 20). **f** Proline contents of KY131, OE and CRI lines in shoots at the two-leaf stage rice seedlings after 45 °C treatment for 48 h. Values are the means± SE, *n* = 3. Differences between the KY131 and transgenic lines were analyzed with Student’s *t*-test. (*P < 0.05; **P < 0.01). **g** DAB staining of KY131, OE and CRI lines leaves from plants under normal (left) and stressed (right, 45 °C) conditions, respectively

To investigate the biological function of *OsProDH* in abiotic stress, we generated *OsProDH* overexpression (OE) lines and knockout mutant lines using CRISPR/Cas9 (CRI) system under KY131 background. Two OE and CRI lines were chosen and characterized in detail for this study (Fig. 3a, b). In CRI-1 and CRI-2 mutants, there was a G and T insertion in the second exon, respectively, resulting in frameshift, which led to a truncated protein (453 aa) and mutant protein that is disrupted at Leu202 and lacks the proline dehydrogenase domain (Fig. 3b and Additional file 2: Figure S3).

Based on gene annotation and domain analyses, we conclude that *OsProDH* encodes proline dehydrogenase. To validate this conclusion, we determined the proline contents of OE and CRI lines and KY131. Results revealed that proline contents of the two CRI lines were highest, followed by that of KY131, and then those of the two OE lines (Fig. 3c). These results were consistent with the annotation results.

We then investigated the biological function of *OsProDH* in seedlings exposed to drought, salt and heat stresses. Under drought and salt conditions, no obvious phenotypic difference was detected between transgenic lines and KY131 (data not shown). However, under high temperature stress conditions, CRI and OE lines were respectively more resistant and sensitive to heat stress than KY131 (Fig. 3d). Specifically, the survival rates were higher in CRI lines and much lower in OE lines than compared with that in KY131 (Fig. 3e). These results indicate that OsProDH negatively regulate thermotolerance in rice seedlings.

Previous studies have reported that environmental stresses such as drought (Choudhary et al. 2005), salinity (Yoshiba et al. 1995), high light and UV irradiation (Saradhi et al. 1995), heavy metals (Schat et al. 1997), and oxidative stress (Yang et al. 2009) can induce proline accumulation in higher plants. Moreover, proline accumulation in plants has a protective function under stress conditions (Kishor et al. 2005; Verbruggen and Hermans 2008). Therefore, we compared the proline contents of wild type KY131 and transgenic seedlings (OE and CRI lines) under 45 °C treatment for 48 h. The results show that proline contents of all seedlings under heat stress were greater than those of the seedlings under the normal condition (Fig. 3c, f). Further, similar to normal conditions, proline contents of CRI and OE lines were significantly higher and lower than proline content of KY131, respectively (Fig. 3f).

Abiotic stress induces ROS accumulation and excessive ROS leads to programmed cell death (Gill and Tuteja 2010). Several studies have revealed that proline exhibits scavenging activity for reactive oxygen species (ROS) and acts as a singlet oxygen quencher (Smirnoff and Cumbes 1989; Matysik et al. 2002). These previous discoveries led us to compare the H_2_O_2_ levels of KY131 and transgenic seedlings under heat stress. We used 3, 3′-diaminobenzidine (DAB) staining to visually evaluate H_2_O_2_ accumulation in leaves. The brown precipitate indicative of H_2_O_2_ accumulation was generally not distributed in the leaves of both KY131 and transgenic lines prior to the heat treatment (Fig. 3g). However, after treatment, we observed more precipitate present in OE leaves than in those of KY131, and more precipitate present in KY131 leaves than those in CRI (Fig. 3g). Taken together, these data suggest that mutation of *OsProDH* led to greater proline accumulation which reduced H_2_O_2_ accumulation and oxidative stress, ultimately conferring higher survival rates despite the heat treatment. Our study provides robust evidence supporting potential genetic approaches to improve crop thermotolerance by engineering proline metabolism.

## Supplementary information

**Additional file 1.** Materials and Methods.

**Additional file 2: Figure S1.** Gene structure and domain annotation of OsProDH. **Figure S2.** Multiple sequence alignment of OsProDH, AtProDH1, AtProDH2, ZmProDH1, ZmProDH2 and SbProDH. The blue lines indicated the conserved proline dehydrogenase domain. **Figure S3.** Characterization of mutation in OsProDH. Protein sequences of OsProDH in KY131 and mutants (CRI-1 and CRI-2) derived from the CRISPR-Cas9 system.

**Additional file 3: Table S1.** Primers used in this study were listed.

## Data Availability

The datasets supporting the conclusions of this article are included within the article and its additional files.

## Abbreviations

KY131: Kongyu131
CDS: Coding sequence
OE: Overexpression
CRI: CRISPR/Cas
GSA: Glutamate-semialdehyde
P5CS: Pyrroline-5-carboxylate synthetase
P5CR: Pyrroline-5-carboxylate reductase
ProDH: Proline dehydrogenase
P5CDH: Pyrroline-5-carboxylate dehydrogenase
