# Supplementary material for: OsProDH Negatively Regulates Thermotolerance in Rice by Modulating Proline Metabolism and Reactive Oxygen Species Scavenging
Source: Rice (N Y). 2020 Aug 26;13:61. doi: 10.1186/s12284-020-00422-3 (PMC7450016; doi:10.1186/s12284-020-00422-3)
Supplement: Supplementary file 1 — Additional file 1. Materials and Methods. [file 12284_2020_422_MOESM1_ESM.docx]

**Materials and Methods**

**Plant materials and heat stress treatment**

For heat treatment, rice plants including KY131, OE and CRI lines were hydroponically cultured in Kimura's culture solution B with the following composition: 0.18 mM (NH_4_)_2_SO_4_, 0.27 mM Mg(SO_4_)_2_, 0.091 mM KNO_3_, 0.091 mM KH_2_PO_4_, 0.046 mM K_2_SO_4_, 0.18 mM Ca(NO_3_)_2_, and 0.04 mM EDTA-Fe, and grown under normal conditions (28 ℃). Then, two-leaf stage seedlings were incubated at 45 °C for 48 hour in light incubator and they were returned to normal conditions (28 °C) for recovery.

**Vector construction and transgenic experiments**

For overexpression test, the full-length coding sequence of *OsProDH* was cloned and ligated into the binary vector pZH2Bi. For CRISPR/Cas9 system, the plant expression vector was constructed as previously described (Endo et al. 2019). All plant expression vectors were introduced into KY131 by *Agrobacterium tumefaciens*-mediated transformation. Primers used for vector construction were listed in Additional file 3: Table S1.

**RNA extraction and quantitative RT-PCR analysis**

Total RNA was extracted using TaKaRa MiniBEST Plant RNA Extraction Kit. The first-strand cDNA was synthesized from 1μg total RNA according to the manufacturer’s protocol (Promega, Madison, USA). qPCR was performed using the SYBR Green I Master (DBI) with Bio-Rad CFX96 machine according to the manufacturer’s protocol. Three repeats were carried out for each gene. For normalization, the *OsActin* gene was used as the internal control. Primers used for qPCR were listed in Additional file 3: Table S1.

**OsProDH subcellular localization**

The *OsProDH* coding sequence was cloned in frame with the GFP under *CaMV 35S* promoter. For confocal microscopy, the localization patterns of OsProDH-GFP was examined by rice protoplasts using a confocal laser scanning microscope (Leica TCS SP5). Preparation and transformation of rice protoplasts were performed according to previous method with minor modifications (Zhang et al. 2011). GFP was excited at 488-nm wave length, and the emission filters were 500–530 nm.

**Proline content measurement and 3,3' -diaminobenzidine (DAB) staining**

For proline content measurement, the shoots of two-leaf stage seedlings were sampled for measurement. Proline content was determined as described previously (Kavi et al. 1995). DAB staining was carried out by the method of Guo et al. (2014).

**References**

Endo A, Saika H, Takemura M, Misawa N and Toki, S (2019) A novel approach to carotenoid accumulation in rice callus by mimicking the cauliflower *Orange* mutation via genome editing. Rice 12: 81.

Guo M, Wang R, Wang J, Hua K, Wang Y, Liu X, Yao S (2014) ALT1, a Snf2 family chromatin remodeling ATPase, negatively regulates alkaline tolerance through enhanced defense against oxidative stress in rice. PLoS One 9: e112515.

Kavi Kishor PB, Hong Z, Miao G, Hu CA, Verma DPS (1995) Overexpression of Δ-pirroline-5-carboxylate synthetase increases proline production and confers osmotolerance in transgenic plants. Plant Physiol 108: 1387–1394.

Zhang Y, Su J, Duan S, Ao Y, Dai J, Liu J, Wang P, Li Y, Liu B, Feng D, Wang J, Wang H (2011) A highly efficient rice green tissue protoplast system for transient gene expression and studying light/chloroplast-related processes. Plant Methods 7: 30.
