# Supplementary material for: OsProDH Negatively Regulates Thermotolerance in Rice by Modulating Proline Metabolism and Reactive Oxygen Species Scavenging
Source: Rice (N Y). 2020 Aug 26;13:61. doi: 10.1186/s12284-020-00422-3 (PMC7450016; doi:10.1186/s12284-020-00422-3)
Supplement: Supplementary file 2 — Additional file 2: Figure S1. Gene structure and domain annotation of OsProDH. Figure S2. Multiple sequence alignment of OsProDH, AtProDH1, AtProDH2, ZmProDH1, ZmProDH2 and SbProDH. The blue lines indicated the conserved proline dehydrogenase domain. Figure S3. Characterization of mutation in OsProDH. Protein sequences of OsProDH in KY131 and mutants (CRI-1 and CRI-2) derived from the CRISPR-Cas9 system. [file 12284_2020_422_MOESM2_ESM.docx]

**
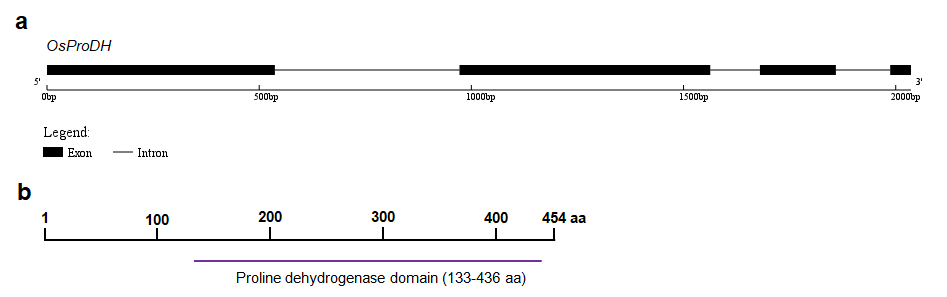
**

**Figure S1.** Gene structure and domain annotation of OsProDH.

**OsProDH    MAIASRIQ---KRV--LASFAAAAAAKLPEAAVAAAGGAAEAVEE------VASSVQEQVQAQGAQVLEFGD**
**AtProDH1   MATRLLRTNFIRRSYRLPAFS---PVGPPTVT-ASTAVVPEILSFGQQAPEPPLHH-PKPTEQSHDGLDLSD**
**AtProDH2   MANRFLRPNLI---HRFSTVS---PVGPPTTI------IPEILSF----------D-QP---KPEVDLDLSD**
**ZmProDH1   MAIASRAT---KRA--LSTFA---AAKLPEEAVAAAAAG-EAIAARSHQA-VPLPP--APAS-SVRPLQFED**
**ZmProDH2   MAIASRAT---KRA--LSTFA---AAKLPEEAIAATAAV----------P-VPLAP--APASSERALLQFED**
**SbProDH    MAIASRVT---KRA--LSTFA---AAKLPEEAVAAAAAG-EAIAATTAAA-VPLASSERTAIARAALLQFED**
**cons       **              :.:.:   ..  *                                      *:: ***


**OsProDH    TERLFAGERSTSLVRTLAVLQALSVGPLVDVATAALRSPAVAGSAA---GRAAARATAYQHFCAGETAEEAA**
**AtProDH1   QARLFSSIPTSDLLRSTAVLHAAAIGPMVDLGTWVMSSKLMDASVTRGMVLGLVKSTFYDHFCAGEDADAAA**
**AtProDH2   QARLFASVPISTLLRSTAILHATSIGPMVDLGSWLMSSKLMDTTVTRDLVLRIVKGTFYDHFCAGEDAAAAA**
**ZmProDH1   TGRLFAGEPTSALLRTLAALQALSVGPLVDAATAALRSPAVAGSAL---GRAAARATAYRHFCAGETADEAA**
**ZmProDH2   TGRLFAGEPTPALLRTLAALQALSVGPLVDAATAALRSPAVAGSAL---GRAAARATAYRHFCAGETAGEAA**
**SbProDH    TGRLFAGEPTSALLRTLAALQALSVGPLVDAATAALRSPAVAGSAL---GRAAARATAYRHFCAGETADEAA**
**cons         ***:.   . *:*: * *:* ::**:** .:  : *  :  :.        .:.* * ****** *  ****


**OsProDH    AAVRRLWR-GGMGGILDYGIEDAEDGPACDRNAAGFLAAIDVAAALPPGS-ASVCIKITALCPVALLEKASD**
**AtProDH1   ERVRSVYEATGLKGMLVYGVEHADDAVSCDDNMQQFIRTIEAAKSLPTSHFSSVVVKITAICPISLLKRVSD**
**AtProDH2   RRVSSVYESTGLKGMLVYGVEHAEDGGACDENIQKFIETVEAAKTLPSSHLSSVVVKITAICPMNVLKRVSD**
**ZmProDH1   AVVRRLWR-GGMGGILDYGIEDAEDGHACDRNAAGFVSAVDVAASLPPGS-ASVCIKITALCPIALLEKASD**
**ZmProDH2   AAVRRLWR-GGMGGILDYGIEDAEDGGACDRNAAGFASAVDVAAKLPPGS-ASVCIKITALCPIALLEKASD**
**SbProDH    AVVRRLWR-GGMGGILDYGIEDAEDGDACDRNAAGFISAVDVAAALPPGS-ASVCIKITALCPIALLEKTSD**
**cons         *  ::.  *: *:* **:*.*:*. :** *   *  :::.*  **..  :** :****:**: :*::.****


**OsProDH    LLRWQQKHPATKLPWKVHGFPVLCVSSPLYLTAAEPPALEAEEERELEMAHGRLLAIGERCAEYDIPLLVDA**
**AtProDH1   LLRWEYKSPNFKLSWKLKSFPVFSESSPLYHTNSEPEPLTAEEERELEAAHGRIQEICRKCQESNVPLLIDA**
**AtProDH2   LLRWQYKNPNFKLPWKLNSFPVFSGLSPLYHTTSEPEPLTVEEERELEKAHERLKSVCLRCQESNVPLLIDA**
**ZmProDH1   LLRWQKKHPSFSLPWKTHSFPVLSDSSPLHLTASEPAALTAEEERELQLAHSRLLAVCARCAEHGIPLLVDA**
**ZmProDH2   LLRWQKRHPSLNLPWKTHDFPILSDSSPLHLTASEPPALSAEEERELELAHERVLAVCARCAERGVPLLVDA**
**SbProDH    LLRWQKKHPSFNLPWKTHSFPILSDSSPLHLTPSEPPALTSEEETELQLAHERLLAVCARCAEHGIPLLVDA**
**cons       ****: : *  .*.** :.**::.  ***: * :** .*  *** **: ** *:  :  :* * .:***:****


**OsProDH    EYATVQPAIDYFTFAGALAFNG-------GGRPIVHGTVQAYLRDARDRLEAMARAAQGERVCLALKLVRGA**
**AtProDH1   EDTILQPAIDYMAYSSAIMFNAD------KDRPIVYNTIQAYLRDAGERLHLAVQNAEKENVPMGFKLVRGA**
**AtProDH2   EDTILQPAIDYMAYWSAIMFNSD------KDRPIVYNTIQAYLKDAGERLHLALRESEKMNVPIGFKLVRGA**
**ZmProDH1   EYATVQPAIDYFTLVGALAFNDAG-AAD-GGRPIVHGTIQAYLRDARDRLEAMVRGAERERVRLGLKVVRGA**
**ZmProDH2   EYAAVQPAIDYLTLAGALACNA--------ERSIVHGTVQAYLRDARERLETMARGVERARVRLGVKLVRGA**
**SbProDH    EYATVQPAIDYFTFVGALAFNDGAGAGDCEQRPIVHGTIQAYLRDARDRLEAMVRSAERERVRLGLKVVRGA**
**cons       * : :******::  .*:  *          *.**:.*:****:** :**.   :  :  .* :..*:******


**OsProDH    YLAREARLAASLGVPSPVHRSIQDTHDCYNGCAAFLLDR---------------------------------**
**AtProDH1   YMSSEASLADSLGCKSPVHDTIQDTHSCYNDCMTFLMEKASNGSGFGVVLATHNADSGRLASRKASDLGIDK**
**AtProDH2   YMSSEAKLADSLGYKSPVHDTIQNTHDCYNDCMSFLMEKASNGSGIAVILATHNTDSGKLGARKASELGINK**
**ZmProDH1   YLTREARLAAALGVPSPVHGSIQETHDCYNGCAAFLLDRVRRGSA-SVMLATHNVESGQLAAARAQELGIPK**
**ZmProDH2   YLAREARVAAALGVPSPVHGSIRETHDCYNGCAAFLLDRVRRGSA-SVVLATHNVESGQLAAARAEELGIPR**
**SbProDH    YLARETRLAATLGVPSPIHGSIQETHDCYNGCAGFLLDRVRRGTA-SVMLATHNVESGKLAAARAQELGIPR**
**cons       *:: *: :* :**  **:* :*::**.***.*  **:::**


**OsProDH    ---GLQFAQLMGMADGLSLGLRNAGFQVSKYLPYGPVEQIIPYLIRRAEENRGLLSSSSFDRQLLRKELVRR**
**AtProDH1   QNGKIEFAQLYGMSDALSFGLKRAGFNVSKYMPFGPVATAIPYLLRRAYENRGMMATGAHDRQLMRMELKRR**
**AtProDH2   ENGKIEFAQLYGMSDALSFGLKRAGFNVSKYMPYGPVDTAIPYLIRRAYENRGMMSTGALDRQLMRKELKRR**
**ZmProDH1   GDRNLQFAQLMGMADGLSLSLRNAGFQVSKYLPYGPVEQIIPYLIRRAEENRGLLSASSFDRQLLRKELVRR**
**ZmProDH2   GDRNLQFAQLMGMADGLSLGLRNAGFQVSKYLPYGPVEQIVPYLIRRAEENRGLLSASSFDRQLLREELVRR**
**SbProDH    GDRNLQFAQLMGMADGLSLSLRNAGFQVSKYLPYGPVEQIIPYLIRRAEENRGLLSASSFDRHLLRKELVRR**
**cons           ::**** **:*.**:.*:.***:****:*:***   :***:*** ****::::.: **:*:* ** ****

**OsProDH    FKAAML--GRE**
**AtProDH1   LIAGI-----A**
**AtProDH2   VMAW-------**
**ZmProDH1   VKAMVA--GRE**
**ZmProDH2   FKAAVL--GRE**
**SbProDH    VKTAVVGMGRE**
**cons       . :**

**Figure S2.** Multiple sequence alignment of OsProDH, AtProDH1, AtProDH2, ZmProDH1, ZmProDH2 and SbProDH. The blue lines indicated the conserved proline dehydrogenase domain.

KY131   MAIASRIQKRVLASFAAAAAAKLPEAAVAAAGGAAEAVEEVASSVQEQVQAQGAQVLEFGDTERLFAGERSTSLV
CRI-1   MAIASRIQKRVLASFAAAAAAKLPEAAVAAAGGAAEAVEEVASSVQEQVQAQGAQVLEFGDTERLFAGERSTSLV
CRI-2   MAIASRIQKRVLASFAAAAAAKLPEAAVAAAGGAAEAVEEVASSVQEQVQAQGAQVLEFGDTERLFAGERSTSLV
cons    ***************************************************************************

KY131   RTLAVLQALSVGPLVDVATAALRSPAVAGSAAGRAAARATAYQHFCAGETAEEAAAAVRRLWRGGMGGILDYGIE
CRI-1   RTLAVLQALSVGPLVDVATAALRSPAVAGSAAGRAAARATAYQHFCAGETAEEAAAAVRRLWRGGMGGILDYGIE
CRI-2   RTLAVLQALSVGPLVDVATAALRSPAVAGSAAGRAAARATAYQHFCAGETAEEAAAAVRRLWRGGMGGILDYGIE
cons    ***************************************************************************

KY131   DAEDGPACDRNAAGFLAAIDVAAALPPGSASVCIKITALCPVALLEKASDLLRWQQKHPATKLPWKVHGFPVLCV
CRI-1   DAEDGPACDRNAAGFLAAIDVAAALPPGSASVCIKITALCPVALLEKASDLLAV---------------------
CRI-2   DAEDGPACDRNAAGFLAAIDVAAALPPGSASVCIKITALCPVALLEKASDLLSV---------------------
cons    ****************************************************                       

KY131   SSPLYLTAAEPPALEAEEERELEMAHGRLLAIGERCAEYDIPLLVDAEYATVQPAIDYFTFAGALAFNGGGR-PI
CRI-1   -------AAEAPGDEAAMESA------------------RVPG-----AVRLQPAVPDG--GGAAGAGGGGGEGA
CRI-2   -------AAEAPGDEAAMESA------------------RVPG-----AVRLQPAVPDG--GGAAGAGGGGGEGA
cons           ***.*. **  *                     :*       . :***:     .** . .***    

KY131   VH--------------------GTVQAYLRDARDRLEAMARAAQGERVCLALKL-VRGAYLAREARLAASLGVPS
CRI-1   RDGARAAAGDRRAVRGVRHPAAGGRRVRHRAAGDRLLHVRRRAGVQRRREAHRARHRPGLPPRRARPAGGHGA--
CRI-2   RDGARAAAGDRRAVRGVRHPAAGGRRVRHRAAGDRLLHVRRRAGVQRRREAHRARHRPGLPPRRARPAGGHGA--
cons     .                    *  :.  * * ***  : * *  :*   * :   * .  .*.** *.. *.  

KY131   PVHRSIQDTHDCYNGCAAFLLDRGLQFAQLMGMADGLSLGL----------------------------------
CRI-1   -------------SGAGRARVPRAQAGPRRVPGARGPPRGLPRRAVAGPPQHPGHPRLLQRLRRVPPRPRPAVRA
CRI-2   -------------SGAGRARVPRAQAGPRRVPGARGPPRGLPRRAVAGPPQHPGHPRLLQRLRRVPPRPRPAVRA
cons                 .*..   : *.   .: :  * * . **                                  

KY131   ---------------RNAGFQVSKYLPYGPVEQIIPYLIRRAEENRGLLSSSSFDRQLLRKELVRRFKAAMLGRE
CRI-1   ADGHGGWPLARPPQRRVPGEQVPAVRSSG-ADHPVPHQTSRGEQGIALVF-------------LLRQTAAPEGAC
CRI-2   ADGHGGWPLARPPQRRVPGEQVPAVRSSG-ADHPVPHQTSRGEQGIALVF-------------LLRQTAAPEGAC
cons                   * .* **.   . * .:: :*:   *.*:. .*:              : * .**  *  

KY131   ----------
CRI-1   EEVQGCNAGT
CRI-2   EEVQGCNAGT
cons

**Figure S3.** Characterization of mutation in OsProDH. Protein sequences of OsProDH in KY131 and mutants (CRI-1 and CRI-2) derived from the CRISPR-Cas9 system.
