# Supplementary material for: OsProDH Negatively Regulates Thermotolerance in Rice by Modulating Proline Metabolism and Reactive Oxygen Species Scavenging
Source: Rice (N Y). 2020 Aug 26;13:61. doi: 10.1186/s12284-020-00422-3 (PMC7450016; doi:10.1186/s12284-020-00422-3)
Supplement: Supplementary file 3 — Additional file 3: Table S1. Primers used in this study were listed. [file 12284_2020_422_MOESM3_ESM.docx]

**Table S1.** Primers used in this study were listed.

| **Primers used for qPCR** | | **Description** |
| --- | --- | --- |
| *OsProDH(RT)-*F | ATCAGACGAGCAGAGGAGAA |  |
| *OsProDH(RT)-*R | CAGCATTGCAGCCTTGAAC |  |
| *OsActin-*F | TGACGGAGCGTGGTTACTCATTCA |  |
| *OsActin-*R | TCTTGGCAGTCTCCATTTCCTGGT |  |
| **Primers used for transgenic construction** | |  |
| OE-F | TCTAGAATGGCCATCGCCTCCCGCATC | Overexpression |
| OE-R | CCCGGGTCACTCACGTCCCAGCATTGCAG |  |
| GFP-F | CCATGGATGGCCATCGCCTCCCGCATC | OsProDH-GFP |
| GFP-R | CCATGGTCCCTCACGTCCCAGCATTGCAG |  |
